# Effect of average volume-assured pressure support treatment on health-related quality of life in COPD patients with chronic hypercapnic respiratory failure: a randomized trial

**DOI:** 10.1186/s12931-020-1320-7

**Published:** 2020-03-06

**Authors:** Doaa M. Magdy, Ahmed Metwally

**Affiliations:** Department of Chest Diseases, Faculty of Medicine, Assuit University, Assiut University Hospital, Assuit, 71515 Egypt

**Keywords:** Health status, Pulmonary disease, Chronic obstructive/epidemiology, Quality of life

## Abstract

**Abstract:**

The long-term effect of average volume-assured pressure support (AVAPS) on health-related quality of life (HRQOL) in chronic obstructive pulmonary disease (COPD) patients with chronic hypercapnic respiratory failure (CHRF) remains unclear.

The objective of this study is to identify the long-term effect of AVAPS in COPD patients with CHRF through assessment of HRQOL, exercise tolerance after six months duration.

**Methods:**

In this randomized, controlled, parallel-group study, 40 stable hypercapnic COPD patients were randomized in a 1:1 ratio to receive either spontaneous timed AVAPS (ST/AVAPS) (intervention) or Bilevel positive airway pressure (ST/BiPAP) (control). HRQL was measured with the Short Form 12 Health Survey Questionnaire (SF-12). Exercise tolerance assessed by 6 min walking distance. Analyses were done between groups from baseline to the average of six months measurements.

**Results:**

AVAPS led to significant 6 months improvements in several domains of (SF-12) compared to the control group, with the greatest improvement seen in general health [treatment effect of 8.2 points (95% confidence interval [95% CI 3.2 to 11.7; *p* = 0.001)], vitality (treatment effect 5.4 points [95% CI 1.4 to 9.3]; *p* = 0.001), physical functioning 5.5 points [95% CI 1.1 to 9.8]; *p* = 0.001) and bodily pain 5.1 points [95% CI 3.4 to 8.8]; *p* = 0.002). The physical health summary score improved by 3.7 points (95% CI 1.2 to 5.8; *p* = 0.001), but no significant improvement in the emotional or social role functioning, mental health subscale was noted.

AVAPS also resulted in improvement 6 min walking distance 9.2 points (95% CI − 1 to − 15];*p* = 0.001). A significant reduction in the daytime (PaCO2) was observed after 6 months in those treated with AVAPS.

**Conclusions:**

In COPD patients with hypercapnic respiratory failure, AVAPS improved exercise tolerance and multiple domains of HRQOL over six months of follow-up, with the significant improvement observed in general health.

## Background

Chronic obstructive pulmonary disease (COPD) is a chronic disease with higher mortality and morbidity worldwide. Patients with end-stage COPD frequently develop chronic hypercapnic respiratory failure (CHRF) associated with end-of-life. In this stage of the disease, patients experience extremely disabling symptoms of dyspnoea, increased risk for frequent exacerbations, re-hospitalization leading to severely impaired health-related quality of life (HRQOL), with limited treatment options [1].

Several factors predispose severe COPD patients to chronic respiratory failure; severe airflow obstruction, hyperinflation, imbalances in the respiratory muscle length-tension relationship, malnutrition, constant use of systemic steroid, and comorbid conditions. Hence, chronic nocturnal non-invasive positive pressure ventilation (NiPPV) could provide respiratory muscle rest, thus enhancing recovery from chronic respiratory muscle fatigue and thereby improving respiratory muscle strength, improving gas exchange [2].

Evidence to support interventions for the treatment of chronic respiratory failure in COPD, except for the use of long-term oxygen therapy (LTOT), has been lacking. NiPPV is commonly instituted in COPD patients with hypercapnia during hospitalization for acute exacerbations and subsequently continued as an outpatient sporadically. However, the evidence to support this intervention is conflicting with no consistent benefit in survival, need for re-hospitalization, clinical impact, quality of life, sleep efficiency or 6 min walk distance [3].

Average volume-assured pressure support (AVAPS) is one of the forms of (NiPPV) that uses automated algorithms to adjust pressure support (PS) to deliver appropriate preset target ventilation to stabilize the PaCO_2_, which relates directly to alveolar ventilation over several breaths [4].

AVAPS is an advanced technology of ventilation designed to smooth patients’ comfort and prevent any potential di-synchronization. AVAPS come to revolutionize the way Bi-Level therapy provided to the breathing complication among patients. Only a few studies have been done to compare the effectiveness and safety of bilevel positive airway pressure (BiPAP) to AVAPS in chronic respiratory failure secondary to obesity hypoventilation syndrome, obstructive sleep apnea, chronic obstructive pulmonary disease, and neuromuscular disorders with respiratory muscle weakness [5]..

Recently published studies have shown positive results with AVAPS in COPD patients, indicated by normalization or reduction of PaCO2 [5, 6]. While there is no doubt that AVAPS improves blood gas and lung function outcomes in COPD patients, there has been conflicting evidence for its benefits on health-related quality of life and survival.

The purpose of this study was to attempt to determine the positive outcomes of AVAPS in stable hypercapnic COPD patients through assessment of daytime PaCO_2_ levels, exercise tolerance, the effect on the quality of life measures.

## Patients and methods

This randomized parallel study was conducted from February 2018 to November 2019 Assiut University Hospital. The study was approved by the Faculty of Medicine Ethics Committee, Assiut University. Informed consent was obtained from the patients.

All Patients with stable COPD and chronic hypercapnic respiratory failure with an indication for chronic NiPPV were enrolled in this study.

Inclusion criteria were: (1) COPD stage III or IV according to GOLD guidelines [1] (post-bronchodilator Forced Expiratory Volume in 1 s (FEV1)/Forced Vital Capacity (FVC) < 70%. FEV1 < 50% of predicted); (2) daytime PaCO_2_ at room air> 45 mmHg in stable condition, defined as no COPD exacerbation during the last 4 weeks, and a pH > 7.35; (3) age > 18 years; (4) existence of a sufficient social support for initiation NPPV at home and (5) written informed consent.

Exclusion criteria were: (1) unstable cardiac condition (unstable angina, arrhythmia); (2) Non-adherent with NPPV treatment (usage < 4 h/night), alterations in mental status (3) Other chronic respiratory diseases (obesity hypoventilation, overlap syndrome, etc.).

**Fig. 1:**
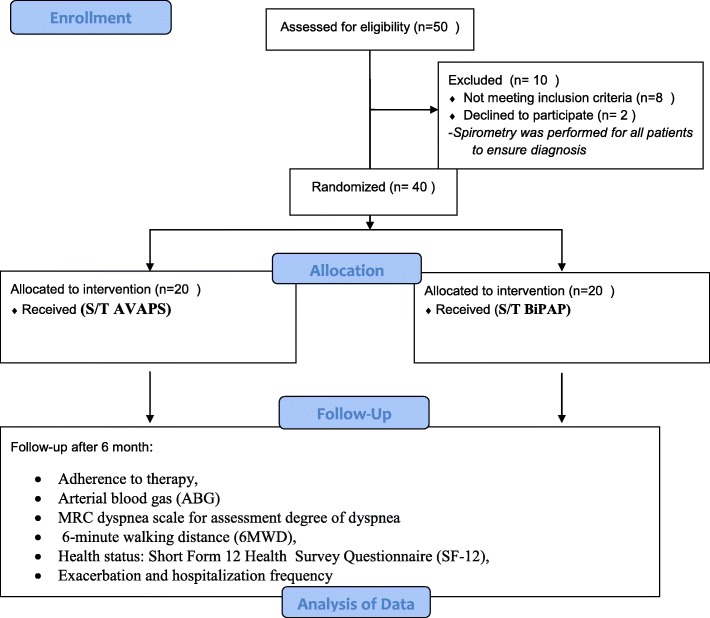
Flow chart diagram

We programmed to include 20 patients in each group; patients divided into (Group I) with ST/AVAPS mode (Intervention) and (group II) with ST/BiPAP mode (Control). Patients eligible for the study were randomized using the random assignment technique formally prepared by a computer-generated program (Fig. 1).

- The *primary outcome* was to assess the change in daytime PaCO_2_ measured during spontaneous breathing at room air after six months compared with baseline.
- *Secondary outcomes* were adherence (average PAP therapy use of ≥4 h per night) – health-related quality of life (HRQOL), measured by (Short Form 12 Health Survey Questionnaire (SF-12), the Medical Research Council score to assess dyspnea; exercise tolerance determined by 6 min walking distance, exacerbation and hospitalization frequency (estimated by checking medical records from the hospital and the general practitioner and by checking taken medication for oral corticosteroid and/or prescribed antibiotics for COPD exacerbations)

### Data collection

All demographic data were collected in a single visit; in which the following variables were recorded: age; sex; Body mass index (BMI); smoking habit (current smoker, never smoker, ex-smoker); year of COPD diagnosis; COPD therapy were evaluated including nonpharmacological management (smoking cessation counselling, stop smoking treatment, oxygen therapy, rehabilitation programs) and pharmacological treatment (long-acting β2-agonists, short-acting anticholinergics, long-acting anticholinergics, inhaled steroids, a fixed combination of anticholinergics and short-acting β2-agonists, a fixed combination of long-acting β2-agonists and inhaled steroids, antibiotics, others).

Lung function was assessed according to international guidelines by spirometry (forced expiratory volume in 1 s (FEV1), forced vital capacity (FVC)) [7].

An assessment degree of dyspnea was obtained according to the **Medical Research Council (MRS) dyspnea scale** [8]**.** The MRC dyspnea scale consists of five degrees: 1, “shortness of breath with strenuous exercise”; 2, “shortness of breath when hurrying”; 3, “walking slower than people of the same age on the level ground or stop for breath while walking at own pace on the level ground”; 4, “needing to stop after 100 yards on the level ground”; 5, “too breathless to leave the house”.

- 6-min walking distance (6MWD) [9] was performed to determine exercise tolerance according to the European Respiratory Society/American Thoracic Society guidelines, along with a 30 m indoor with standardized encouragements given by the physician.
- Arterial blood gas (ABG) analysis using an automated analyzer was obtained. (Model 850, Chiron Diagnostics, Medfield, MA) (pH, PaCO2, PaO2)
- Health status: Short Form 12 Health Survey Questionnaire (SF-12) [10]:

The SF-12 is a widely used and validated self-reported instruments used for assessing HRQOL. The SF-12 questionnaire consists of twelve questions that measure eight health domains for evaluating physical and mental health status.

Physical health-related domains include General Health (GH), Physical Functioning (PF), Role Physical (RP), and Body Pain (BP). Mental health-related scales include Vitality (VT), Social Functioning (SF), Role Emotional (RE), and Mental Health (MH). Each health domain is scored on a 0 to 100 metric, the higher score reflecting better health.

We administered the SF-12 monthly by telephone survey for all participants through the 6-month duration. Scores from the eight domains, and the two derived summary scores (physical component summary (PCS) and mental component summary scores (MCS) were recorded and analyzed.

#### NiPPV initiation

Patients who met inclusion criteria were allocated on a1:1 basis by computer-generated allocation numbering using a random sequence to S/T AVAPS or S/T BiPAP.

Spontaneous/timed -Average volume-assured pressure support **(S/T AVAPS):**

Ventilatory parameters were initially programmed in the S/T AVAPS mode (Respironics trilogy 202 ventilator, Philips). Initial ventilator settings were: Patient’s height in cm, Target alveolar ventilation (Va): adjusted provided that tidal volume is 6–8 ml/kg of ideal body weight (IBW), Expiratory Positive Airway Pressure (EPAP):4–8 cmH_2_O, minimum and maximum pressure support (PS): 8–16, Respiratory rate: 15 breath/min. As the operator sets the target tidal volume (ml/kg IBW) and the target minute ventilation, the equivalent target alveolar ventilation is calculated -based on the patient’s introduced height using a special formula that calculates dead space ventilation where: Dead Space Ventilation (VD) =120x(h/175)^2.363^ . Subtracting the dead space ventilation from the target minute ventilation, alveolar ventilation is obtained and displayed, where: VA = MV-VD.

So, the operator can make sure VA matches the patient’s ventilatory demands [5].

Spontaneous/timed- Bilevel positive airway pressure (**S/T BiPAP):**

Ventilatory parameters were programmed in S/T BiPAP mode (Respironics trilogy 202 ventilator, Philips), including IPAP at 12 cmH_2_O, and EPAP at 4–8 cmH_2_O. The respiratory rate was set at 15 breaths/min, rise time set at 300–400 ms, and inspiratory time set at a minimum of 0.6 s. IPAP was measured in increments of 2 cmH_2_O according to the discretion of the attending physician.

Supplemented O_2_ was added through an adapter circuit close to the facemask to maintain SaO_2_ (oxygen saturation) above 90%.

### Follow-up

All patients visited the outpatient clinic 3 months (limited assessment) and 6 months (full evaluation) after the NiPPV was initiated and follow-up measurements were performed by one of the investigators, who were not blinded to the allocation sequence. Patients could contact us by telephone whenever they had any questions.

### Statistical analysis

A per-protocol analysis was performed, including all patients who were compliant with their NiPPV and who completed the study. Safety analyses were performed on all randomized patients who received either S/T AVAPS or S/T BiPAP that had at least a measurement at baseline and after 6 months.

Analyses were performed using SPSS for Windows (version 22.0, Chicago, Illinois, USA). Parametric data were described using mean and standard deviation and comparisons made using paired *t-* tests or unpaired *t-* tests, as appropriate; otherwise, non-parametric equivalents were used. For all analyses, a *p*-value of < 0.05 was considered statistically significant.

Differences in baseline variables between the S/T AVAPS and S/T BiPAP were tested with a t-test or Mann-Whitney U test for continuous variables and χ2 tests for categorical variables. A general linear repeated measures analysis of variance with Bonferroni correction or a paired t-test was performed.

We calculated the absolute change in outcome variables (SF-12 Health Survey Questionnaire, MRC dyspnea scale and 6MWD from baseline to 6 months and performed a linear regression analysis with correction for the baseline value calculating the adjusted mean difference between the groups.

## Results

A total of 40 patients with stable COPD (stage III or IV patients) and chronic hypercapnic respiratory failure were screened as outpatients and randomized when they met all inclusion criteria, and subsequently planned for NiPPV initiation either with ST/AVAPS) *(intervention) and ST/BiPAP (control)group.* All participants in the treatment group were compliant to AVAPS or BiPAP for an average of ≥4 h/night over 6 months.

The sociodemographic and clinical characteristics of the patients are shown in Table 1.

**Table 1:** Sociodemographic and clinical characteristics of patients

	Patients treated with AVAPS (*N* = 20)	Control group (*N* = 20)
Age, years	65.8 ± 9.8	65.4 ± 8.4
Sex: Male n%)	12 (60%)	10 (50%)
Body mass index (BMI)	24.8 ± 8.4	25.4 ± 7.9
Smoking		
• Never	1(5%)	0(0%)
• Ex-smoker	5(25%)	6 (30%)
• Current smoker	14 (70%)	14(70%)
Years since COPD diagnosis	9.5 ± 7.4	9.7 ± 7.9
FEV1, L	0.60 ± 0.16	0.59 ± 0.21
FVC, L	2.19 ± 0.55	1.90 ± 0.50
Underlying disease		
Diabetes	7(35%)	5(25%)
Hypertension	6 (30%)	8(40%)
Dyslipidemia	3 (15%)	4(20%)
Chronic kidney disease	1 (5%)	2 (10%)
Coronary artery disease	4 (20%)	3(15%)

*FEV1* forced expiratory volume in 1 s*, FVC* forced vital capacity

### Gas exchange

Over the 6 months follow-up period, AVAPS led to a more significant reduction in daytime PaCO_2_. The estimated treatment effect was 0.6 points (95% CI − 0.3 to 0.9) between the AVAPS versus the control group. Other parameters of gas exchange were also significantly improved between groups (Table 2).

**Table 2:** Gase exchange

Variables	Patients treated with AVAPS		Control group		Treatment Effect (95% CI) 6 months–baseline	*P*- Value
	Baseline	After 6 month	Baseline	After 6 month		
Pa CO_2_ mm Hg	54.8 ± 3.5	46.6 ± 3.1	54.5 ± 3.3	48.3 ± 3.9	0.6 (−0.3 to 0.9)	0.001*
PaO_2_ mm Hg	50.7 ± 2.1	59.6 ± 2.3	50.9 ± 2.5	57.7 ± 3.2	0.6(−0.67 to 0.56	0.001*
HCO_3_^−^, mmol/L	34.2 ± 3.1	29.5 ± 2.1	34.9 ± 3.2	30.6 ± 2.9	0.4(−1.4 to 1.6)	0.001*

*Data are shown as mean ± SD. A positive mean difference means an increase from baseline to 6 months for AVAPS compared with control group. HCO*_*3*_^*−*^ *= bicarbonate; PaCO2, partial arterial carbon dioxide pressure; PaO2, partial arterial oxygen pressure*

### Outcomes

After 6 months of treatment, AVAPS improved several domains of the SF-12 compared to the control group. The greatest effect was observed for general health, with an estimated treatment effect of 8.2 points (95% confidence interval [CI] 3.2 to 11.7; *p* = 0.001) comparing the AVAPS to the control group. Significant improvements were also seen for vitality (estimated treatment effect 5.4 points [95% CI 1.4 to 9.3]; *p* = 0.001), physical functioning 5.5 points [95% CI 1.1 to 9.8]; *p* = 0.001) and bodily pain 5.1 points [95% CI 3.4 to 8.8]; *p* = 0.002).

The physical health summary score improved by 3.7 points (95% CI 1.2 to 5.8; *p* = 0.001), but no significant improvement in the emotional or social role functioning, mental health subscale, or summary score was noted. The degree of dyspnea was improved in those treated with AVAPS, although no statistical significance was reached. However, AVAPS resulted in improvement 6 min walking distance (mean change in 6MWD 9.2 point [95% CI − 1 to − 15];*p* = 0.001). (Table 3).

**Table 3:** Patient-reported outcomes (SF-12, MRC scale, and 6 MWD)

Variables	Patients treated with AVAPS		Control group		Treatment Effect (95% CI) 6 months–baseline	*P*-Value
	Baseline	After 6 month	Baseline	After 6 month		
SF-36 Scales *#*						
Vitality	55.2 ± 16.1	64.3 ± 17.5	56.1 ± 17.3	60.5 ± 16.2	5.4(1.4 to 9.3)	0.001*
General health	56.3 ± 20.5	61.3 ± 16.7	57.6 ± 19.2	58.4 ± 20.2	8.2(3.2 to 11.7)	0.001*
Physical functioning	72.4 ± 23.8	77.5 ± 21.5	69.3 ± 24.2	69.6 ± 22.1	5.5(1.1 to 9.8)	0.001*
Bodily pain	62.3 ± 23.2	65.5 ± 22.4	63.7 ± 23.6	66.7 ± 20.8	3.1 (3.4 to 8.8	0.002*
Emotional Role Functioning	80.6 ± 22.5	83.5 ± 24.7	79.0 ± 24.8	82.3 ± 20.7	−0.5 (−6.8 to 5.3)	0.321
Physical Role Functioning	69.3 ± 23.3	73.5 ± 26.6	70.8 ± 23.7	73.2 ± 24.8	3.3(−5 to 6.6)	0.001*
Social Role Functioning	79.6 ± 23.8	83.6 ± 24.7	80.8 ± 21.9	82.9 ± 25.8	1.6 (−3.8 to 6.4)	0.143
Mental health	74.4 ± 18.6	75.8 ± 12.8	75.6 ± 15.7	75.4 ± 15.4	1.2 (−1.7 to 6.9)	0.543
PCSS	45.3 ± 9.6	51.3 ± 8.4	44.1 ± 8.4	44.7 ± 7.2	3.7 (1.2 to 5.8)	0.001*
MCSS	50.3 ± 10.8	53.5 ± 12.7	49.2 ± 11.8	52.7 ± 11.2	1.1 (−2.7 to 2.9)	0.436
MRC scale *#*	4.3 ± 0.5	3.1 ± 0.2	4.1 ± 0.7	4 ± 0.4	0.4(−0.2 to 3)	0.213
6MWD, m *#*	178.2 ± 24.3	260.5 ± 32.2	179.3 ± 32.1	255.2 ± 30.2	9.2(−1 to 15)	0.001*

*Data presented as mean (standard deviation); #*: *The SF-12, MRC scores, and 6MWD were skewed distributed; therefore a Mann-Whitney U test was performed to compare the changes within the groups; the delta scores were normally distributed, therefore the changes were compared with a regression analysis. A positive mean difference means a decrease or an increase from baseline to 6 months for the S/T AVAPS compared to S/T BiPAP (control)*

*Abbreviations: SF-12* Short Form 12 Health Survey Questionnaire *CI* confidence interval *PCSS* physical component summary score *MCSS* mental component summary score, *MRC* Medical Research Council *6MWD* 6-min walking distance

### Exacerbations and hospitalizations

There was no difference in change in the number of hospital days, hospitalization, and exacerbation frequency comparing the 6 months before inclusion to the study period between the two groups (Table 4).

**Table 4:** Exacerbations and hospitalizations

Variable	Patients treated with AVAPS		Control group		Treatment Effect (95% CI) 6 months–baseline	*P*-value
	6 months before inclusion	Study period (6 months after AVAPS start)	6 months before inclusion	Study period (6 months after BiPAP start)		
Exacerbation (n)	2.7 ± 1.7	1.5 ± 0.3	2.6 ± 1.9	1.5 ± 0.2	−0.9 (− 0.9 to 0.7)	0.213
Hospitalization (n)	3.2 ± 1.1	1.1 ± 0.1	3.5 ± 1.3	1.2 ± 0.1	−0.1 (− 0.6 to 0.4)	0.342
Hospital days(n)	5.2 ± 2.1	3.4 ± 2.1	5.3 ± 2.2	3.5 ± 2	− 1.5 (− 5 to 2)	0.321

*number of exacerbations, hospitalizations and hospital days data*

## Discussion

COPD Patients with chronic hypercapnic respiratory failure; experience respiratory symptoms and physical incapacity that negatively influence health status, which itself is a predictor of frequent hospitalizations and higher mortality [11].

Hence, the principal purpose of the study is to assess the impact of AVAPS treatment in COPD patients with CHRF on health status; through a structured interview incorporates the Short Form 12 (SF-12) over a 6-month duration. The SF-12 has been exhibited to be a valid evaluation tool that enables to calculate the physical well-being and the mental-well being components of HRQOL.

The main findings in this study, treatment with AVAPS, led to significant 6- month improvements in several domains of HRQOL, with the most significant increase seen in the general health domain. The effect is stronger on the physical than the mental aspects of health status. Prior studies in COPD patients with stable hypercapnic respiratory failure have demonstrated that patients receiving home-based NiPPV reported more restful sleep when using AVAPS as compared with conventional NiPPV [12–15]. However, up to date, no study has consistently addressed the benefit of AVAPS on HRQOL.

Another important consideration in this study is adherence to therapy during the long-term use of AVAPS. In one study, Kelly et al. reported that AVAPS led to one additional hour per night of use compared with conventional PS [5]. Such improvements in adherence could have an impact on patient outcomes [16].

Concerning gasometric parameters, we compared ABG values (PaCO_2_, Pa O_2_, and HCO3) at baseline and after 6 months of AVAPS treatment. We observed overall ABG alterations in both groups but a significant reduction in PaCO_2_ after 6 months in the AVAPS treated group. Crisafulli et al. previously reported improved gas exchange in hypercapnic COPD patients who received AVAPS [6]. Moreover, a recent randomized trial [17] comparing patient satisfaction between (ST/BiPAP with AVAPS) and ST/BiPAP alone in 22 COPD patients; has shown that overall comfort and patient satisfaction was higher in the AVAPS group as an adjunct to standard BiPAP S/T therapy (1.64 ± 2.77 vs. 1.09 ± 3.02). Besides, they found a greater decrease in blood pressure, heart rate, and blood gases with S/T AVAPS group compared with the ST/BiPAP group. Therefore, COPD patients might benefit from the theoretical advantage of a guaranteed tidal volume during nocturnal ventilation**.**

In our study on COPD patients, it appears noteworthy that the frequency of exacerbation and hospitalization days was reduced in response to AVAPS treatment, although statistical significance was not reached. Thus, these results potentially suggesting a more favourable added benefit if used for a more extended period.

The strengths of this study include its randomized design and the use of the SF-12 questionnaires to assess health status. The SF-12 is one of the most frequently used and valid instrument to evaluate HRQOL.

However, limitations of the present study are; the number of patients was minimal, that the results cannot be generalized to COPD patients with exacerbation, as all included patients were initiated at least 4 weeks after an exacerbation. Therefore, we think that larger sample size and a longer follow up period should be done to test our hypothesis.

## Conclusion

In this study in COPD with resting hypercapnia respiratory failure, the use of AVAPS demonstrated a significant effect on reducing PaCO2, exercise tolerance, and improving quality of life measures during the 6-months of daily use. Future studies are needed to delineate the optimal candidate for AVAPS and the most useful settings to provide optimal ventilation to enhance patient compliance.

## Data Availability

The datasets used and analyzed during the current study are available from the corresponding author on reasonable request.

## Abbreviations

6MWD: 6- min walking distance
AVAP: Average volume-assured pressure support
CHRF: Chronic hypercapnic respiratory failure
CI: Confidence interval
COPD: Chronic obstructive pulmonary disease
FEV1: Forced expiratory volume in 1 s
FVC: Forced vital capacity
HCO_3_^−^: Bicarbonate
HRQOL: Health-related quality of life
MCSS: Mental component summary score
MRC: Medical Research Council
NiPPV: Non-invasive positive pressure ventilation
PaCO2: Partial arterial carbon dioxide pressure
PaO2: Partial arterial oxygen pressure
PCSS: Physical component summary score
S/T AVAPS: Spontaneous/timed -Average volume-assured pressure support
SF-12: Short Form 12 Health Survey Questionnaire
ST/BiPAP: Spontaneous/timed Bilevel positive airway pressure
